# Welfare Work in the Malay States

**Published:** 1924-11

**Authors:** 


### WELFARE WORK IN THE MALAY STATES.

As a supplement to a recent number of the *F.M.S. Government Gazette* there has been published a "Report on Infant Welfare and Maternity Work in Kuala Lumpur for the past year." The work, started in 1922, and carried on in two rooms of a Chinese dwelling-house, has two branches—an Infant Welfare Centre and a Health Visiting organisation. The staff consists of a lady medical officer, a superintendent nursing sister (European), and two health visitors (locally trained). By degrees the prejudice against the invasion of the home by stranger health visitors is lessening, and that the work is becoming increasingly popular will be seen by the fact that the attendances grew from 50 a month in July, 1922, to over 1,000 a month in June, 1923.

### The Propaganda Exhibition.

Occasionally fathers come for instruction; one Sikh father said that his wife was not clever and that he had come to learn in her stead. There is an Ante-Natal Clinic which advises expectant mothers and diagnoses and treats venereal diseases in women. The Advisory Board and Committee for Public Health Education issues pamphlets and posters in Malay, Tamil, and Chinese, with a view to familiarising the public with the objects of the service. Its most valuable propaganda work during the year was an Infant Welfare Exhibit at the Malayan Agri-Horticultural Exhibition at Kuala Lumpur in July. This exhibit was visited by large numbers of Asiatics of all classes, and the extent to which the work is appreciated was shown by the fact that prominent representatives of both the Chinese and the Tamil communities requested that arrangements should be made for a permanent exhibit.

### Medical Advice on Athletics.

An addition to Spalding's Athletic Library (Penwick, 180 Fleet Street. 1s. net.) is a thoroughly sensible little book on "Hygiene and Happiness," by Major Leonard Tosswill, M.R.C.S., L.R.C.P., late Assistant M.O. to the Public Health Department of the L.C.C. Major Tosswill thinks such races as the quarter mile "particularly dangerous to the growing boy," calling not only for violent, but also for prolonged effort. For the long compulsory run in favour at some public schools he has only condemnation.

